# Interactions between HIV-1 and the Cell-Autonomous Innate Immune System

**DOI:** 10.1016/j.chom.2014.06.009

**Published:** 2014-07-09

**Authors:** Greg J. Towers, Mahdad Noursadeghi

**Affiliations:** 1 Division of Infection and Immunity, University College London, London W1CE 6BT, UK

## Abstract

HIV-1 was recognized as the cause of AIDS in humans in 1984. Despite 30 years of intensive research, we are still unraveling the molecular details of the host-pathogen interactions that enable this virus to escape immune clearance and cause immunodeficiency. Here we explore a series of recent studies that consider how HIV-1 interacts with the cell-autonomous innate immune system as it navigates its way in and out of host cells. We discuss how these studies improve our knowledge of HIV-1 and host biology as well as increase our understanding of transmission, persistence, and immunodeficiency and the potential for therapeutic or prophylactic interventions.

## Teaser

HIV-1 relies on complex host interactions to complete its life cycle, but also to evade antiviral defenses. Towers and Noursadeghi discuss recent progress in understanding how HIV-1 interacts with the cell-autonomous innate immune system and how this knowledge could lead to therapeutic opportunities and a better understanding of HIV disease.

## Main Text

### The Cell-Autonomous Innate Immune System

Like all viruses, lentiviruses parasitize their hosts and rely on a complex interaction with host intracellular functions to complete their life cycles. HIV-1 has evolved to recruit host dependency factors, or cofactors, as well as evade or manipulate the cell-autonomous innate immune system that has evolved over millions of years to defend the host from infection. This defensive system provides a mechanism by which cells can detect the presence of a pathogen and also deploy a series of local and systemic defensive measures that enhance and mediate antiviral defenses. Importantly, the cell-autonomous innate immune system also influences the adaptive immune system, providing information on the nature of the pathogen and thus the appropriate adaptive response. The molecular tripwires of the cell-autonomous innate immune system are pattern recognition receptors (PRRs) that recognize pathogen-associated molecular patterns (PAMPS) within the specific compartments that they patrol. In this respect, principal cellular PRRs can be broadly classified as transmembrane receptors of the Toll-like receptor (TLR) family that sense extracellular or endosomal compartments. There is also an increasing repertoire of cytoplasmic receptors, mainly with specificity for pathogen nucleic acids as well as nonmicrobial danger-associated molecular patterns.

**Figure 1:**
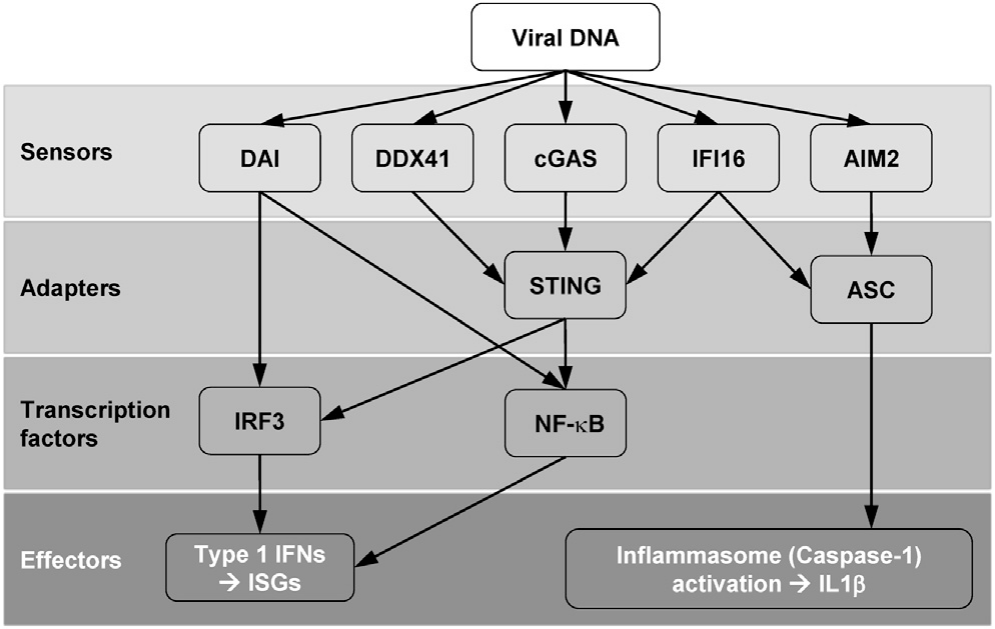
Innate Immune IFN Responses and Caspase-1-Mediated Activation of Cytokines or Cell Death Are Functionally Coupled by Upstream PRRs for Viral DNA Sensors: cGAS, cGAMP synthase; IFI16, IFN-inducible protein 16; DAI, DNA-dependent activator of IRFs; DDX41, DEAD Asp-Glu-Ala-Asp box polypeptide 41. Adapters and transcription factors: ASC, activating signal cointegrator 1; STING, stimulator of IFN gene; NF-κB, nuclear factor kappa-light-chain-enhancer of activated B cells; IRF3, IFN regulatory factor 3.

The canonical response to PRR stimulation leads to activation of signaling cascades and, typically, nuclear translocation of cytoplasmic transcription factors exemplified by NF-κB RelA and IRF3 with consequent transactivation of innate immune response genes. In the antiviral response, this is dominated by induction and secretion of soluble type 1 interferon (IFN). This leads to autocrine, paracrine, or endocrine activation of cell surface IFN receptors and downstream intracellular JAK/STAT signaling cascade activation (Stark and Darnell, 2012), resulting in a second line of gene expression changes that lead to development of the so-called antiviral state. The antiviral state is mediated by the combination of all the genes induced by the IFN response and can comprise hundreds of proteins whose expression is increased by exposure to IFN. In this way both infected and nearby uninfected cells become nonpermissive to viral replication through expression of a host of diverse antiviral activities that lead to the suppression of replication of most viruses as well as other pathogens. In fact, the ability to antagonize or evade this response is probably the most important determinant for viral replication, or tropism, in a particular host. Importantly, PRRs also lie upstream of the inflammasome, in which caspase activation can unleash the function of inflammatory procytokines and initiate apoptosis or pyroptosis pathways, thereby coupling innate immune sensing of pathogens to host cell death (Figure 1).

Although this robust IFN response is initiated, until recently the suppression of HIV-1 replication by IFN and the characterization of how HIV-1 avoids triggering IFN responses have been understudied. This is partly because IFN induces the expression of many effector genes, making the system rather complicated to dissect. However, studies examining the mechanisms underlying species-specific replication of lentiviruses as well as the role of lentiviral accessory proteins have led to excellent progress in this field. This work uncovered IFN-stimulated factors that restrict HIV, notably APOBEC3G (Sheehy et al., 2002), TRIM5α (Stremlau et al., 2004), tetherin (Neil et al., 2008; Van Damme et al., 2008), SAMHD1 (Hrecka et al., 2011; Laguette et al., 2011), and more recently Mx2 (Goujon et al., 2013; Kane et al., 2013). Many studies have shown that HIV-1 is sensitive to IFN (Tsang et al., 2009), but it is only now becoming possible to dissect the IFN effector systems using modern high-throughput molecular approaches. For example, comparative gene expression arrays were used to identify both tetherin (Neil et al., 2008) and Mx2 (Goujon et al., 2013; Kane et al., 2013). Here, we discuss recent progress in understanding how HIV-1 interacts with the cell-autonomous innate immune system and how this new knowledge could lead to new therapeutic opportunities for viral infection and a better understanding of HIV disease.

### Cytoplasmic DNA Sensors that Detect HIV-1 Infection

Because retroviruses reverse transcribe their RNA genome into double-stranded DNA in the cytoplasm of infected cells, innate DNA sensors that detect cytoplasmic DNA pose a particular problem for this family of viruses. Cytoplasmic DNA sensors have received a great deal of attention recently. As a result, we are beginning to understand the details of their sensing mechanisms and the downstream consequences of their activation. IFI16 is a cytoplasmic DNA sensor thought to have a role in detection of specific viruses, including HIV-1 (Jakobsen et al., 2013; Monroe et al., 2014), whose life cycle involves DNA in the cytoplasm of infected cells (Unterholzner et al., 2010). However, the recently identified DNA sensor cGAS has generated considerable attention with regard to HIV-1 infection. This sensor synthesizes a second messenger molecule formed from cyclic AMP and cyclic GMP called cGAMP (Wu et al., 2013), giving rise to the name cGAMP synthetase, or cGAS, for the sensor (Sun et al., 2013). The cGAMP produced by cGAS binds to the adaptor molecule STING, leading to dimerization of the transcription factor IRF3 and activation of IRF3-regulated genes (Figure 1). Evasion of DNA sensors is thought to be particularly important for lentiviruses, including HIV-1, not only because they synthesize DNA in the cytoplasm of infected cells, but also because they are particularly sensitive to the effects of IFN. In most cases, lentiviruses are strongly repressed in cells treated with type 1 IFN (Goujon and Malim, 2010; Tsang et al., 2009). We therefore believe that HIV-1 is highly evolved to avoid triggering DNA sensors, using a process we have termed cloaking.

### Evasion of DNA Sensors by Cloaking

**Figure 2:**
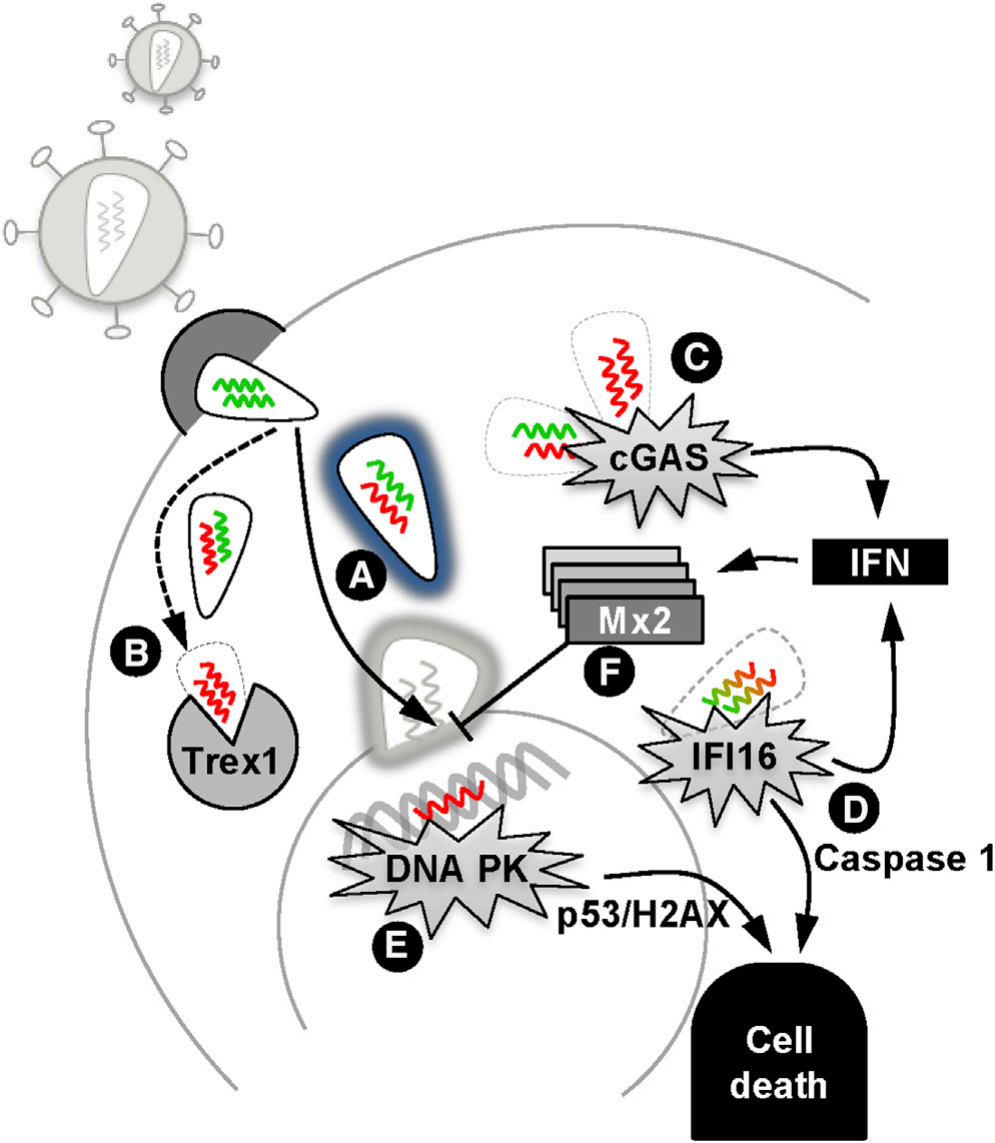
The Relationship between HIV-1, DNA Sensors, and IFN Production Determines Successful Replication versus Abortive Infection and Cell Death (A–F) In macrophages, recruitment of host factors (CPSF6 and CypA) to the HIV-1 capsid, shown as a blue border (A), and degradation of cytoplasmic viral DNA by the exonuclease TREX1 (B) prevents IFN responses and allows HIV-1 to establish productive infection. Mutations in capsid or small molecule inhibitors that prevent binding of CypA lead to IFN responses due to cGAS-mediated detection of the DNA products of HIV-1 reverse transcription (RT) even in the presence of TREX1 (C). In T cells, the sensing of incomplete RT products by IFI16 led to activation of the inflammasome and caspase-1-dependent cell death as well as stimulation of the IFN responses (D). Cell death in T cells has also been attributed to a DNA protein kinase (DNA PK)-dependent DNA damage response that involves p53 and histone H2AX and results from incomplete HIV-1 DNA integration events (E). Mx2 is an exemplar for the antiviral effectors that are induced after sensing and that restrict viral infection. Mx2 restricts HIV-1 nuclear entry, and no viral countermeasures are known (F). Mx2 does not fully account for IFN-mediated restriction of wild-type HIV-1, suggesting the presence of further, yet-unidentified restriction factors.

In a series of studies from several labs, HIV-1 has been shown to utilize cellular cofactors that allow evasion of DNA sensor activation and thereby prevent triggering of innate responses. In the first example, the endoplasmic reticulum-associated nuclease TREX1 was shown to be a critical cofactor for HIV-1 replication (Yan et al., 2010) (Figure 2). At first, it seems paradoxical that a nuclease that degrades HIV-1 DNA could act as a cofactor for infection. However, in the absence of TREX1, excess cytoplasmic DNA produced by reverse transcription appears to trigger the cytoplasmic DNA sensor cGAS, leading to production of the second messenger cGAMP (Gao et al., 2013; Sun et al., 2013; Yan et al., 2010). cGAMP production leads to type 1 IFN production, via STING activation, and suppression of replication. Thus, HIV-1 appears to have evolved to synthesize DNA in such a way that TREX1 degrades excess reverse transcription products and prevents the virus from triggering cytosolic DNA sensors. In individuals that are defective for TREX1, through genetic polymorphism, an autoinflammatory encephalitis manifests, called Aicardi-Goutieres syndrome. This debilitating disease is characterized by high levels of IFN production, thought to be due to activation of DNA sensors by endogenous viruses (Stetson et al., 2008). Thus, paradoxically, despite acting as a nuclease and degrading viral DNA, TREX1 acts as a viral cofactor, preventing HIV-1-mediated triggering of innate DNA sensors and promoting viral replication. In this way, TREX1 negatively regulates activation of cell-autonomous innate immunity, potentially to the advantage of both endogenous and exogenous retroviruses. We assume that the evolutionarily advantageous rearrangement of genes provided by retrotransposition has led to TREX1-mediated tolerance of cytoplasmic reverse transcription. Although this pathway has evolved for use by the relatively unrelated, reverse-transcribing, endogenous retroelements, exogenous retroviruses such as HIV-1 can exploit this tolerance.

### HIV-1 Cloaking in Macrophages

**Figure 3:**
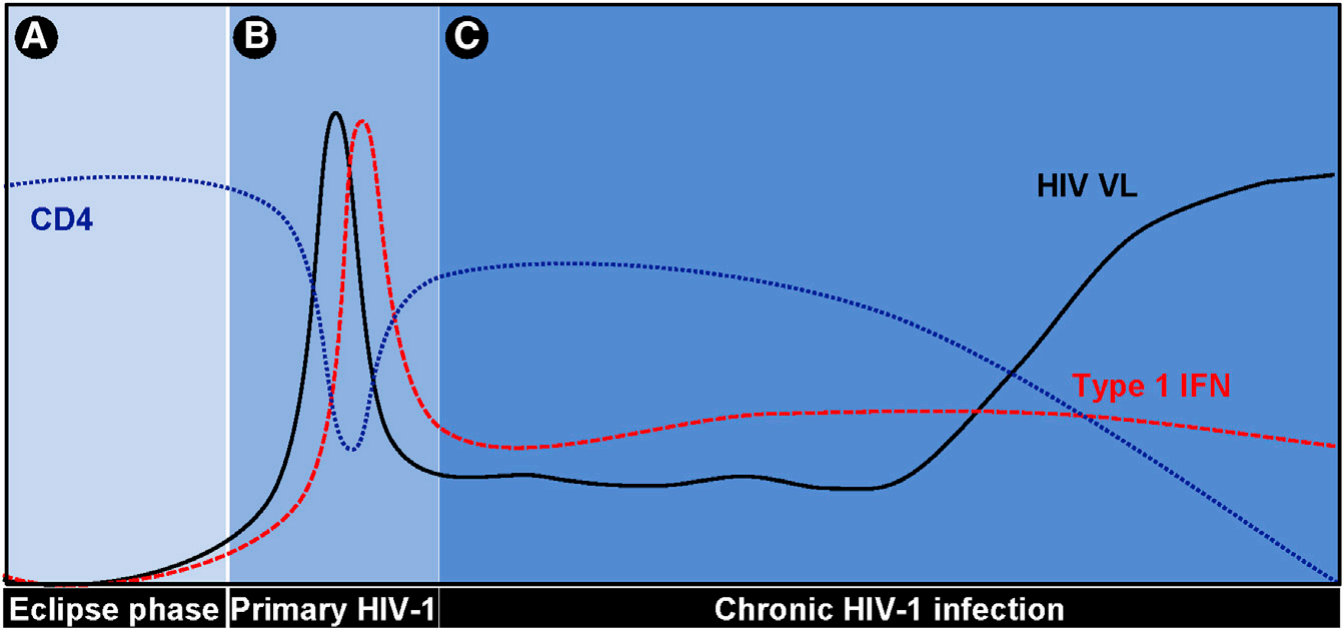
Type 1 IFN May Have Positive and Negative Effects Requiring Different Therapeutic Strategies at Each Stage of the Course of HIV-1 Disease (A–C) Changes to CD4-positive T cells (CD4), type 1 IFN, and HIV-1 viral load (HIV VL) are represented schematically over the course of HIV-1 infection. We propose that HIV-1 goes under the radar of innate immune detection and hence evades IFN restriction in the eclipse phase that follows virus inoculation (A), allowing it to establish a foothold in host cells. The viremia that then arises from massive virus propagation in T cells is associated with significant T cell death and a systemic type 1 IFN response (B). Innate immune responses that link induction of IFN and activation of the inflammasome may contribute to the control of viremia, but also mediate T cell death by pyroptosis. Chronic IFN responses associated with persistent viremia may contribute to progressive HIV-1 disease associated with chronic immune activation (C). The different roles for innate immune responses to HIV-1 at each stage may offer specific therapeutic opportunities, such as enhancing innate immune detection by uncloaking the virus to reduce transmission efficiency, targeting caspase-1 activation to reduce T cell death without compromising IFN-mediated restriction during primary HIV-1 disease, and inhibiting HIV-1 induction of IFN in chronic infection to attenuate progressive disease.

We recently suggested that HIV-1 can replicate in human macrophages without triggering innate sensors because, in addition to TREX1, the virus has evolved to utilize several additional cellular cofactors to cloak its presence and avoid detection by PRRs (Rasaiyaah et al., 2013) (Figure 2). HIV-1 recruits two particular cofactors, cyclophilin A (CypA) and CPSF6, to the incoming capsid (Franke et al., 1994; Lee et al., 2010; Price et al., 2012; Towers et al., 2003). CypA is an abundant cytoplasmic prolyl isomerase enzyme that recruits to the surface of the incoming HIV-1 capsid and isomerizes the peptide bond between capsid residues G89 and P90. CPSF6 also recruits to the HIV-1 capsid while it is in the cytoplasm. CPSF6 is a predominantly nuclear protein with a role in 3′-end mRNA processing, which may contribute to targeting HIV-1 into a particular pathway of nuclear entry (Rasaiyaah et al., 2013). These cofactors are critical for HIV-1 replication in primary human monocyte-derived macrophages (MDM) (Rasaiyaah et al., 2013). When interactions between CA and CypA or CPSF6 are prevented, such as by mutating the viral capsid, HIV-1 triggers DNA sensors and activates a type 1 IFN response, leading to suppression of replication. Specifically, the HIV-1 capsid mutant N74D cannot recruit CPSF6 and the P90A mutant capsid cannot recruit CypA. HIV-1 encoding either of these mutations cannot replicate in MDM due to simulation of type 1 IFN. The importance of IFN in suppressing replication is illustrated by the fact that blockade of IFN signaling using an IFN receptor antibody can rescue the replication defect of the mutant viruses. The DNA sensor cGAS appears to have a role in this process, as increased cGAMP was detected after infection of MDM with the P90A capsid mutant. Similar levels of IFN secretion were elicited after RNAi-mediated CPSF6 depletion and subsequent wild-type virus infection or after prevention of CypA recruitment with a nonimmunosuppressive cyclosporine, CsA-SmBz. This pharmacological uncloaking of HIV-1 suggests a new paradigm for the treatment or prevention of viral infection in which viruses are revealed to innate immune sensors by targeting cloaking cofactors (Figure 3).

The observation that CPSF6 mutants with a defective nuclear localization signal become cytoplasmic and can act as dominant negatives that suppress HIV-1 reverse transcription (Hori et al., 2013; Rasaiyaah et al., 2013) suggested that HIV-1 utilizes CPSF6 to suppress premature DNA synthesis that would otherwise trigger cytoplasmic DNA sensors. Why TREX1 is not able to degrade the viral DNA responsible for DNA sensor triggering in this case remains unclear, but may be due to the quantity, location, or nature of the DNA products. For example, it is plausible that TREX1-dependent DNA clearance is saturated, or that the DNA products form specific immunostimulatory motifs (single- or double-stranded or even RNA/DNA hybrids), which are resistant to TREX1 degradation. The role of CypA is less clear than CPSF6, but it may have a role in camouflaging the hexameric lattice of HIV-1 capsid or an allosteric role in CPSF6 recruitment. In summary, we hypothesize that HIV-1 recruits CypA and CPSF6 to suppress premature reverse transcription and allow evasion of DNA sensors in MDM (Hilditch and Towers, 2014). Importantly, both CypA and CPSF6 were unnecessary for HIV-1 replication in HeLa and HOS cell-based indicator cell lines. This observation suggests that some cell lines may not have intact DNA sensor pathways and may therefore be misleading when examining cofactor dependence of HIV-1 or sensitivity to innate immune sensors.

### HIV-1 Uncoating and Reverse Transcription Determine Cloaking

Early work characterizing the behavior of HIV-1 cores upon target cell entry suggested that the viral cone-shaped capsid containing the reverse transcription complex uncoats in the cytoplasm as a prerequisite to DNA synthesis. However, recent knowledge of DNA sensors and their ability to detect cytoplasmic DNA suggests that HIV may have evolved a more sophisticated uncoating process that can evade DNA sensors, at least in certain circumstances. Microscopic analysis of HIV-1 behavior inside cells suggested that the virus might carry out reverse transcription and uncoating in a highly organized way, perhaps in complex with the nuclear pore, at least in some cells (Arhel et al., 2007). More recent genetic analysis has described multiple direct interactions between the HIV-1 capsid and nuclear pore proteins that might mediate this process. Functional interactions have been described between the viral capsid and nuclear pore components Nup358 (Bichel et al., 2013; Schaller et al., 2011) and Nup153 (Matreyek and Engelman, 2011; Matreyek et al., 2013) and perhaps interaction between integrase and the karyopherin TNPO3 (Christ et al., 2008). Intriguingly, Nup153 is largely found on the inside of the nuclear membrane and yet appears to interact with HIV-1 capsid in the same pocket as CPSF6 (Matreyek and Engelman, 2011; Matreyek et al., 2013). Thus, one possibility is that Nup153 reaches through the nuclear pore complex (NPC) from the nuclear side to displace CPSF6 and uncoat the intact reverse transcribing capsid. Such a model might allow Nup153 to uncoat the viral core in the region associated with the central pore of the NPC. This could allow the DNA to be fed through the pore into the nucleus without being exposed to the cytoplasm at any point, despite being synthesized on the cytoplasmic side of the nuclear membrane. Nuclear entry of the DNA is likely to be immediately followed by integration into chromatin, with nuclear entry and integration occurring as a single coordinated event at the nuclear pore. An attractive aspect of this more complex model of coordinated DNA synthesis, uncoating, nuclear entry, and integration in complex with the NPC is that it suggests a mechanism by which HIV-1 can evade activation of the cell-autonomous innate immune system by avoiding revealing viral DNA to cytoplasmic DNA sensors or free viral DNA ends to the nuclear DNA damage machinery.

### Antagonizing Restriction Factors while Traveling Light

In the case of lentiviruses, such as HIV-1, the IFN-induced effector proteins that are actually responsible for suppressing viral infectivity are called restriction factors. This phrase was coined in the earliest days of considering the tropism of murine leukemia viruses and their suppression by the restriction factor Fv1 (Best et al., 1996). Most restriction factors are expressed at low levels, even in the absence of IFN-mediated induction. These proteins have therefore been described as mediating intrinsic innate immunity because they are intrinsically expressed (Bieniasz, 2004). However, distinguishing between proteins based on their expression levels in uninduced cells may be misleading, and we prefer to think of all IFN-induced proteins as critical and integral features of the cell-autonomous innate immune system.

Lentiviruses travel light, with only 9–10 genes. Because of this, they do not have the genetic capacity to globally manipulate innate immune responses as larger viruses do. For example, herpes viruses encode in excess of 200 open reading frames, the majority of which have roles in antagonizing cellular defensive processes (for review, see Amsler et al., 2013). We hypothesize that the constraints of a small genome provide selective pressure for HIV-1 to evolve evasion strategies, rather than antagonists that abrogate the effector functions of restriction factors. However, because many restriction factors are intrinsically expressed, even in the absence of IFN induction, the virus cannot simply inhibit the IFN pathway but must antagonize or avoid specific restriction factors that pose a barrier to replication. In the case of lentiviruses, accessory proteins carry the burden of restriction factor antagonism. Indeed, it appears that the major function of lentiviral accessory proteins is to act as adaptor molecules that recruit ubiquitination machinery to mark the targeted restriction factor for proteasome-mediated degradation (Schwefel et al., 2014).

The restriction factor tetherin provides a good example of this Red Queen-style antagonistic evolution between the host and virus (Gupta et al., 2009a). Tetherin is a transmembrane protein that forms a physical tether to prevent nascent HIV-1 virions from leaving the surface of infected cells (Neil et al., 2008). HIV-1 is unable to avoid budding through membranes and therefore cannot avoid restriction by tetherin if the protein is present in the host cell plasma membrane. The importance of suppressing tetherin activity is illustrated by the fact that the pandemic HIV-1 strain, HIV-1 M group, has evolved to use the accessory Vpu protein to antagonize tetherin. The parental virus SIVcpz from chimpanzees uses its Nef protein to antagonize tetherin, and the less successful HIV-1 zoonoses, giving rise to HIV-1 groups O, N, and P, have not made this evolutionary transition as effectively and thus antagonize tetherin poorly (Sauter et al., 2009). These observations suggest that tetherin antagonism has been a critical aspect of the evolution of HIV-1 to become pandemic in humans (Gupta and Towers, 2009). All other established primate lentiviruses also have antitetherin activity. Most of the simian immunodeficiency viruses (SIVs), like SIVcpz, use their Nef protein to antagonize tetherin, some use Vpu, and some lentiviruses, notably HIV-2 and SIVtan from Tantalus monkeys, can use their envelope protein for this purpose (Gupta et al., 2009b; Le Tortorec and Neil, 2009). The diversity of viruses restricted by tetherin has led to an equally diverse array of antitetherin activities (for review, see Neil, 2013).

The accessory gene Vif also has an important role antagonizing restriction factors in the shape of the APOBEC3 family of cytidine deaminases (Sheehy et al., 2002). APOBEC3G, as well as several other APOBEC3 proteins, suppresses HIV-1 infectivity by inhibiting DNA synthesis as well as by driving catastrophic hypermutation in any synthesized viral DNA. In this way, the APOBEC proteins are powerful defensive restriction factors, and the absence or failure of Vif to antagonize them via induced degradation leads to potent suppression of viral replication.

SAMHD1 is a restriction factor expressed widely in myeloid cell lineages and resting T cells (Baldauf et al., 2012; Laguette et al., 2011). Restriction by SAMHD1 is mediated by reduction of nucleotide pools to levels where reverse transcription cannot proceed, although the restriction mechanism may be more complex than this simple model (Goldstone et al., 2011; Lahouassa et al., 2012). SAMHD1 appears to be particularly important for protecting dendritic cells from HIV-1 infection (Manel et al., 2010). SAMHD1 does not present a barrier to infection by many SIVs because they encode the accessory protein Vpx, which evolved by duplication of Vpr (Tristem et al., 1992). Vpx antagonizes SAMHD1 by recruiting a ubiquitin ligase complex that leads to SAMHD1 ubiquitination and degradation by the proteasome (Schwefel et al., 2014). HIV-1 does not encode a Vpx homolog and is thus sensitive to restriction by SAMHD1. This sensitivity has an important impact on HIV-1 tropism, with evidence that SAMHD1 prevents HIV-1 infection of dendritic cells and resting T cells (Baldauf et al., 2012; Laguette et al., 2011). HIV-1 is not completely at the mercy of SAMHD1, however, because it can replicate in cells such as macrophages despite the fact they express SAMHD1. Provision of Vpx to HIV-1, either by coinfection with SIV or by manipulating HIV-1 such that it can incorporate the SIV protein (Sunseri et al., 2011), improves HIV-1 infectivity in macrophages, and this confirms a functional role for SAMHD1 in these cells. However, HIV-1 can clearly replicate in macrophages without Vpx. Recent data using a mouse SAMHD1 knockout demonstrated that HIV-1 becomes more sensitive to SAMHD1 in mouse cells if its reverse transcriptase is mutated to have lower dNTP affinity. This observation suggests that wild-type HIV-1 partially escapes SAMHD1 by tolerating lower levels of nucleotides during DNA synthesis (Rehwinkel et al., 2013). Whether this mechanism underpins HIV-1 tropism for macrophages is not yet resolved.

Importantly, if SAMHD1 restriction is experimentally bypassed in human dendritic cells, using SIV Vpx, then HIV-1 is able to reverse transcribe. However, in this case the dendritic cells detect the virus, become activated, and secrete large amounts of type 1 IFN. Dendritic cells are therefore not permissive to a full HIV-1 replication cycle, even when SAMHD1 restriction is suppressed. An initial study suggested that this is because the virus is detected by innate sensors acting after viral integration and detecting a complex between the HIV-1 gag protein and the cellular gag-binding cofactor CypA (Manel et al., 2010). However, a more recent study from this group proposed that, in fact, if SAMHD1 is inactivated with Vpx, then the cytoplasmic DNA sensor cGAS detects HIV-1 reverse transcription products, leading to activation of innate signaling cascades and the maturation of the dendritic cells (Lahaye et al., 2013). This latter model is more consistent with data derived in monocyte-derived macrophages discussed above (Rasaiyaah et al., 2013).

### Mx2 and Beyond

Consistent with the notion of innate evasion being important for HIV-1, it is clear that the virus is unable to antagonize all of the restriction factors that it encounters in the presence of type 1 IFN (Goujon and Malim, 2010; Tsang et al., 2009). This suggests that the repertoire of IFN-induced effector proteins includes yet-unidentified restriction factors with anti HIV-1 activity. The search for new restriction factors active against HIV-1 has recently revealed a role for the GTPase Mx2 in restricting HIV-1 infection in IFN-treated cells (Figure 2). Almost simultaneously, three independent groups identified Mx2 as having activity against HIV-1 (Goujon et al., 2013; Kane et al., 2013; Liu et al., 2013). Mx2 suppresses HIV-1 infectivity after reverse transcription, possibly at the point of nuclear entry. The protein localizes to the nuclear membrane and is therefore well positioned to suppress infection at this stage. Curiously, several HIV-1 capsid mutants, including the CypA (CypA) binding mutant P90A and the CPSF6 binding mutant N74D, have been shown to be insensitive to Mx2 restriction, leading to suggestions of direct interactions between the capsid and Mx2. However, at present, this possibility has not been rigorously explored. Importantly, these HIV-1 capsid mutants are sensitive to IFN treatment despite escaping Mx2, and IFN treatment of most cells leads to a block to HIV-1 reverse transcription rather than nuclear entry (Goujon and Malim, 2010). These observations point to the presence of further host factors capable of suppressing HIV-1 DNA synthesis, and the search for the proteins mediating the anti-HIV-1 effects of IFN remains as competitive as ever.

### Some Innate Immune Effectors Also Signal

One of the most exciting recent developments in the field of lentiviral restriction is the realization that certain prototypic anti-HIV-1 restriction factors can activate an innate response, as well as act as antiviral effector molecules. These factors therefore act as both innate sensors and IFN effectors. Avoiding these proteins is presumed to be particularly important for lentiviruses. This dual feature was described for TRIM5α in a landmark publication in 2011 (Pertel et al., 2011). TRIM5α targets incoming retroviral capsids, which it recruits to proteasomes, shortly after viral entry, leading to a block to reverse transcription and a process that dismantles the virus (Kutluay et al., 2013; Stremlau et al., 2004). However, TRIM5α can also trigger an innate signaling pathway when incoming virions are engaged, acting like a classical PRR (Pertel et al., 2011). The innate signal is dependent on TRIM5α ubiquitin ligase activity, which catalyzes the synthesis of K63-linked ubiquitin chains, leading to activation of TAK1 and NF-κB-dependent signaling pathways. The HIV-1 capsid protein has evolved to be invisible to human TRIM5α and is therefore not restricted by this protein, even when expressed at high levels (Stremlau et al., 2004). Simian TRIM5α proteins, however, have potent anti-HIV-1 activity, and they pose a significant barrier to HIV-1 replication in nonhuman species, particularly in Old World primates.

Like TRIM5α, tetherin also activates an innate signal on engagement with newly formed virions activating an inflammatory signaling that is similar in nature to that elicited by TRIM5α engagement with virus (Galão et al., 2012). The tetherin innate signal is mediated by a tetherin variant expressed from an alternative AUG start codon. Two AUGs allow synthesis of a long and a short tetherin, and while both of these proteins have anti-HIV-1 activity, only the longer protein can generate an innate signal (Cocka and Bates, 2012). Intriguingly, the generation of signaling capacity appears to be a unique property of the human tetherin gene, and this activity is not found in the chimpanzee or simian tetherin proteins. Thus, the ability to generate an innate signal may be a recent adaptation that allows tetherin to signal the presence of a pathogen to surrounding uninfected cells. These observations suggest that in some cases it might be as important for the infected cell to activate the cell-autonomous innate immune response as it is to kill the detected virus. In this way, local cells at the site of infection can be warned of impending infection, and an IFN-induced antiviral state can be established before the virus infection takes hold and becomes systemic. For a small IFN-sensitive virus like HIV-1, antagonizing tetherin is likely to be particularly important to prevent further augmentation of antiviral defenses, particularly during transmission.

### Activation of Sensing by HIV-1 Can Cause Cell Death

Can the relationship between HIV-1 and the cell-autonomous innate immune system help us to understand the pathogenesis of HIV-1? Several recent studies have addressed this question in some detail and have produced some surprising and exciting findings that may have implications for our understanding of HIV biology and the mechanism of immune deficiency. A series of studies seeking to understand why HIV-1 causes T cell death suggest that this relationship is key to understanding HIV-1 disease. The Greene lab has presented several studies that suggest that incomplete reverse transcription products, produced in abortively infected resting T cells, are detected by the DNA sensor IFI16, leading to T cell death by pyroptosis (Doitsh et al., 2010, 2014; Monroe et al., 2014) (Figure 2). The researchers used mechanically disrupted tonsil explants for these studies, reasoning that these primary human tissues would better represent the complexity of T cell subsets and the cytokine milieu found in vivo (Doitsh et al., 2010). They discovered that infection of these cultures with HIV-1 led to productive infection of a small percentage of the activated T cell fraction. Remarkably, infection of this small number of cells led to a profound loss of nonproductively infected T cells, with significant loss of the resting cells that are traditionally thought to be nonpermissive for HIV-1 infection. Loss of uninfected T cells appeared to be dependent on viral fusion and incomplete reverse transcription in the target cell cytoplasm. Killing was most profound when the infected and uninfected cells were in contact, suggesting a role for the viral synapse, although this may simply reflect the increased efficiency of virus transfer in the context of the synapse as opposed to cell-free virus. The abortively infected dying cells displayed activated caspase-1, activation of inflammasomes, and secretion of the potently inflammatory cytokine IL-1β (Doitsh et al., 2010). IL-1β production and activation is tightly controlled, and secretion of bioactive IL-1β requires activation of inflammasomes, which lead to cleavage of the IL-1β precursor by caspase-1 and secretion of the bioactive cytokine. CD4 T cells contain large amounts of IL-1β precursor and are thus primed to activate this inflammatory response. The DNA sensor responsible for caspase-1 activation in abortively infected T cells appears to be IFI16 (Monroe et al., 2014). The authors were able to purify IFI16 from tonsillar CD4 T cell lysates using a 500 bp fragment of HIV-1 DNA as bait. Data supporting a role for IFI16 in the activation of IL-1β production, and the ensuing pyroptosis, were provided by IFI16 depletion experiments using RNAi. IFI16 depletion led to a significant reduction in T cell death after HIV-1 infection as opposed to cultures expressing a control hairpin. Tonsillar T cell cultures depleted for AIM2 or STING were not protected from cell death after HIV-1 infection, suggesting that these factors have no role in the detection of abortive infection and caspase-1 activation. Similar work in monocytes has suggested a role for SAMHD1 in sensing HTLV infection of these cells (Sze et al., 2013).

Importantly, investigation of the causes of T cell death suggests a therapeutic opportunity for caspase-1 activation with specific inhibitors. Indeed, caspase-1 inhibitors have been developed and trialed in anticancer studies and are thus well poised for clinical evaluation in HIV-1-infected patients. At what stage of infection such an intervention may be effective remains open to speculation (Figure 3). Importantly, T cell death by pyroptosis was also reported in cultures of T cells isolated from spleen, suggesting that these findings are not dependent on the source of cells. Furthermore, both CCR5 and CXCR4 viruses were reported to be able to cause pyroptosis and IL-1β production. While these results are yet to be reproduced independently, we cautiously suggest that they may represent a significant breakthrough in our understanding of how HIV-1 replication leads to T cell death and AIDS. An important question that arises is whether SIV infection induces the same effect in T cells purified from a species in which it is nonpathogenic. For example, we might expect that SIVsm would not cause pyroptosis in T cells from Sooty Mangabeys given that this virus does not cause T cell depletion or disease in these animals (Milush et al., 2011).

Work from the Nabel lab has suggested an additional mechanism for cell death after HIV-1 infection (Cooper et al., 2013). In this case, cell death was shown to be due to incomplete integration events, rather than sensing of incomplete reverse transcription products (Figure 2). In this system, cell death involved the triggering of DNA-dependent protein kinase (DNA-PK), a critical component of the DNA damage response, and was observed in a T cell line (CEMX174) as well as primary T cells derived from HIV patient PBMC. Additional studies indicated that the route of viral entry is unimportant for triggering DNA-PK and that neither viral gene expression nor RNA export is required to induce cell death in this model. The integrase inhibitor Raltegravir effectively protected cells from death, consistent with an important role for integration. DNA circularization was not thought to be important in this context because depletion of DNA ligase 4, an enzyme required for DNA circle formation, did not impact cell death, although DNA circles were reduced as expected. Investigating the mechanism of induction of cell death, the authors detected HIV-1 integration-dependent phosphorylation of p53 and histone H2AX. Furthermore, DNA-PK localized to the nuclei of the infected cells. The authors concluded that HIV-1 integration elicits a cellular double-stranded DNA damage response that leads to virus-induced cell death. This process was sensitive to pharmacological inhibition, and DNA-PK inhibitors prevented p53 and H2AX phosphorylation and HIV-1-dependent cell death in both T cell lines and primary CD4+ lymphocyte cultures. Again, these observations require independent reproduction, but they suggest a further possibility for the cause of T cell death induced by HIV-1. Strikingly, it is the defensive processes of the innate immune system that appear to be the cause of cell death in both of these narratives (Figures 1 and 2).

### The Role of Type 1 IFN

Despite the stealth HIV-1 has evolved in its replication in macrophages, it is not capable of universally avoiding triggering IFN production. During primary viremia, HIV-1 causes a wave of infection through the T cell compartment in the mucosal tissues of the gut, and the emergent viremia in primary HIV-1 infection is associated with a cytokine storm (Stacey et al., 2009) that might contribute to some degree of virus restriction, but ultimately fails to sterilize infection (Figure 3). This cascade of inflammatory cytokines includes high levels of type 1 IFN. The prevailing view implicates a role for TLR7-dependent induction of IFN in plasmacytoid dendritic cells (Lepelley et al., 2011) and possibly monocytes (Beignon et al., 2005), and abortively infected T cells may also produce type 1 IFN (Doitsh et al., 2014).

### Are Macrophages Important in HIV-1 Pathogenesis?

Together, the studies described above suggest that HIV-1 may cause more cell death while replicating in T cells than it does when replicating in macrophages. This could be interpreted as HIV-1 being better adapted to replicate in macrophages than in T cells. However, it remains somewhat controversial as to whether macrophages represent a significant target cell type for HIV-1 in vivo. We argue that, while it is undeniably true that the vast majority of HIV-1 replication occurs in T cells, macrophages may nonetheless represent an important target cell type. While almost all the virus in an infected individual is derived from T cells, it seems pertinent that HIV-1 has evolved to replicate in macrophages without triggering cytokine production or cell death. We propose that if it did not avoid triggering IFN responses in these and other myeloid cells, then this would have significant negative consequences on its ability to transmit and replicate to high levels. For example, dendritic cells are not thought to be permissive for HIV-1 replication, yet their infection likely has profound effects on the consequences of HIV-1 infection, and their ability to sense HIV-1 has a critical role in this process (for review, see Luban, 2012). We imagine that HIV-1 behaves differently in different cell types, perhaps due to different cofactor availability or cofactor requirements, and further, that the characterization of the differences and similarities between replication in these cell types will be critical for a full understanding of transmission and pathogenesis as well as the development of new therapeutics and vaccines.

### A Role for IFN in HIV-1 Transmission

Further support for the importance of IFN in inhibiting HIV and thus potentially limiting transmission comes from the study of HIV-1-transmitted founder clones. The viral clones that are responsible for transmitting from one individual to another appear to have been selected for the maximal level of insensitivity to IFN (Fenton-May et al., 2013). High-throughput sequencing of virus from patients at very early stages of infection allows the construction of what are referred to as transmitted founder clones. These virus clones represent the virus that actually transmitted to the patient, and infection can often be shown to be due to a single clone of founder virus (Salazar-Gonzalez et al., 2009). Comparing IFN sensitivity of transmitted founder clones with clones derived from the same patients several years later revealed that the founder clones are significantly less sensitive to IFN than their chronic clone counterparts (Fenton-May et al., 2013). This suggests that the virus becomes more sensitive to IFN between peak viremia and chronicity and is consistent with a selective event during transmission that favors virus that is most insensitive to IFN. Many studies have shown that HIV-1 transmission is a rare event, with many exposures required for each successful event. For example, conservative estimates suggest as many as 100 exposures for each case of HIV-1 infection. An ability to avoid triggering IFN and at the same time be relatively resistant to any IFN that is produced therefore appear to be key features of successfully transmitted viral clones.

We therefore hypothesize that HIV-1 is a virus with the capacity to trigger IFN and a particular sensitivity to IFN, particularly in the mucosa where target cells are more likely to be myeloid than lymphocytic. These features may combine to make transmission a rare event that depends on limited sensitivity to IFN and a quiet approach where innate sensors are concerned. Once transmission has occurred and the virus begins to replicate in activated T cells in the gut, then IFN sensitivity may become less of a problem. We hypothesize that IFN is a good thing for the host at the point of transmission because it induces protective responses. IFN may also be beneficial to the host during primary viremia, where it may help suppress viral replication. However, it seems likely that continuous low-level IFN production during the chronic stage of infection is likely to be detrimental to the host and contribute to disease (Figure 3), for example by promoting TRAIL-mediated bystander apoptosis of CD4 T cells (Barblu et al., 2012; Hardy et al., 2007; Stary et al., 2009). Certainly, prolonged immune activation is associated with lentiviral infections that cause disease over ones that don’t (Evans and Silvestri, 2013). However, our model of IFN-sensitive transmission is an optimistic one. Tipping the balance in favor of IFN and virus suppression should be relatively easy once we have a detailed understanding of HIV-1 evasion strategies and IFN effector mechanisms. Uncloaking the virus pharmacologically could be an effective prophylactic, for example by using nonimmunosuppressive cyclosporines (Rasaiyaah et al., 2013). Route of transmission will be important, of course, and it may be more difficult to prevent infection caused by direct injection of virus or infected cells as is the case with shared needle transmissions. Differences between vaginal and rectal tissues will also influence the effectiveness of any intervention depending on a mucosal environment.

### The Study of SIV in Nonhuman Primates

Lentiviruses are prevalent natural infections of certain African nonhuman primates, but natural infection of monkeys, with their cognate SIV, does not generally lead to significant pathogenicity. The reasons for the lack of disease after natural SIV infection are unclear, but many studies have highlighted high levels of immune activation as a key feature of pathogenic versus nonpathogenic lentiviral infections. Immune activation also strongly correlates with disease in primate models of AIDS in which monkeys, usually Asian macaques, are infected with lentivirus from another species, for example SIVsm lineage viruses. In these cases, infection can lead to an AIDS-like disease very similar to that seen in humans. Natural infection of Sooty Mangabeys with SIVsm, however, does not lead to significant immune activation, and infected animals are largely asymptomatic, despite high viral loads in peripheral blood. The continued study of natural, nonpathogenic, lentiviral infection and its comparison to AIDS in humans and experimentally infected monkeys promises to reveal a great deal of the mechanisms of lentiviral pathogenesis (Evans and Silvestri, 2013). We advise some caution in using SIV to study transmission because the route of SIV transmission is likely to differ from HIV-1, which may have specifically adapted to become a sexually transmitted disease. Comparison between SIV and HIV-1 must also take into account the fact that SIV encodes Vpx, whereas HIV-1 does not, and the tropism for myeloid cells such as dendritic cells that SIV gains through this additional feature.

### Concluding Remarks

The balance between evading host induction of type 1 IFN and suppression of HIV-1 by IFN will be critical to both transmission events as well as subsequent viral replication. A better understanding of how HIV-1 cloaks itself to avoid sensing may help us develop inhibitors of cloaking that can act as powerful prophylactics (Rasaiyaah et al., 2013). Further, discovering the identity of the restriction factors that suppress HIV-1 replication when sensors are activated and IFN is produced will provide important details of the virus’s weak spots, particularly during transmission. We hope that eventually this work will lead to our ability to eradicate the virus from an infected individual and elicit a lasting cure for this obstinate viral pathogen.
